# Structural analysis of the carboxy terminal PH domain of pleckstrin bound to D-*myo*-inositol 1,2,3,5,6-pentakisphosphate

**DOI:** 10.1186/1472-6807-7-80

**Published:** 2007-11-22

**Authors:** Sean G Jackson, Yi Zhang, Richard J Haslam, Murray S Junop

**Affiliations:** 1 Department of Biochemistry and Biomedical Sciences, McMaster University, Hamilton, Canada; 2 Department of Pathology and Molecular Medicine, McMaster University, Hamilton, Canada

## Abstract

**Background:**

Pleckstrin homology (PH) domains are one of the most prevalent domains in the human proteome and represent the major phosphoinositide-binding module. These domains are often found in signaling proteins and function predominately by targeting their host proteins to the cell membrane. Inositol phosphates, which are structurally similar to phosphoinositides, are not only known to play a role as signaling molecules but are also capable of being bound by PH domains.

**Results:**

In the work presented here it is shown that the addition of commercial *myo*-inositol hexakisphosphate (IP_6_) inhibited the binding of the carboxy terminal PH domain of pleckstrin (C-PH) to phosphatidylinositol 3,4-bisphosphate with an IC_50 _of 7.5 μM. In an attempt to characterize this binding structurally, C-PH was crystallized in the presence of IP_6 _and the structure was determined to 1.35 Å. Examination of the resulting electron density unexpectedly revealed the bound ligand to be D-*myo*-inositol 1,2,3,5,6-pentakisphosphate.

**Conclusion:**

The discovery of D-*myo*-inositol 1,2,3,5,6-pentakisphosphate in the crystal structure suggests that the inhibitory effects observed in the binding studies may be due to this ligand rather than IP_6_. Analysis of the protein-ligand interaction demonstrated that this *myo*-inositol pentakisphosphate isomer interacts specifically with protein residues known to be involved in phosphoinositide binding. In addition to this, a structural alignment of other PH domains bound to inositol phosphates containing either four or five phosphate groups revealed that the majority of phosphate groups occupy conserved locations in the binding pockets of PH domains. These findings, taken together with other recently reported studies suggest that *myo-*inositol pentakisphosphates could act to regulate PH domain-phosphoinositide interactions by directly competing for binding, thus playing an important role as signaling molecules.

## Background

Pleckstrin is an intriguing platelet protein that appears to be involved in reorganization of the cytoskeleton, as well as attenuating various signaling pathways following platelet activation [1-6]. On cloning pleckstrin, two internal repeats consisting of approximately 100 amino acids including 30 identical residues were identified at the N- and C-termini of the protein [7,8]. Similar regions were later recognized in other proteins and these internal repeats were consequently termed pleckstrin homology (PH) domains [9,10].

Since their first identification in pleckstrin, PH domains have been found in over four hundred human proteins (SMART database,[11]) making this domain one of the most common in the human proteome. PH domain-containing proteins are known to be involved in a number of different cellular functions, including phosphoinositide metabolism, protein phosphorylation and cytoskeletal organization, suggesting that PH domains themselves may also function in a variety of different ways (reviewed in [12,13]). The crystal and solution structures of numerous PH domains have been determined revealing that despite sharing only low sequence similarity, they maintain a highly conserved fold. The domain structure consists of a seven-stranded anti-parallel β-sandwich that is closed at one end by a C-terminal alpha helix and remains open at the other end, where several variable loop regions are located (reviewed in [13]). Traditionally, PH domains have been thought to function predominately as phosphoinositide-binding modules, targeting their host proteins to the membrane where they can carry out their various functions. The region known to bind these signaling lipids has been identified as the variable β1–β2 loop, with some PH domains contributing additional interacting residues from nearby secondary structure elements. Despite their early characterization as phosphoinositide-binding modules, it is now clear that the majority of PH domains do not bind phosphoinositides with sufficient affinity or specificity to drive membrane localization [12], suggesting that alternate functions are likely to exist for these domains. In support of this notion, several reports have shown that within some proteins, PH domains function to mediate protein-protein interactions [14-16].

Given their wide distribution, it is likely that PH domains mediate other processes in addition to protein-protein interactions and membrane association through phosphoinositide-specific binding. In particular, it seems possible that specific soluble inositol phosphates (such as inositol 1,4,5-trisphosphate[IP_3_]), could bind to PH domains and thereby serve as important regulators [17]. Inositol phosphates are structurally very similar to the phosphoinositide head groups known to bind to many PH domains. In fact, it has been well established that PH domains can bind inositol phosphates *in vitro *and have been used extensively in structural and biochemical studies focused on understanding phosphoinositide-PH domain interactions. Despite this they have received relatively little consideration as physiological PH domain ligands.

Previous studies have shown that phosphatidylinositol 3,4-bisphosphate [PtdIns(3,4)*P*_2_] binds to C-PH much more firmly than other phosphoinositides [18,19] and that IP_6 _is a potent inhibitor of pleckstrin-phosphoinositide interactions [19]. Based on these findings, we found that commercial IP_6 _also competed effectively with PtdIns(3,4)*P*_2 _for binding to C-PH. As we have recently reported the crystal structure of C-PH in the unliganded form [20], we then set out to determine the structural basis for the interaction with IP_6_. To our surprise, we found that the complex formed contained D-*myo*-inositol 1,2,3,5,6-pentakisphosphate [Ins(1,2,3,5,6)*P*_5_], rather than IP_6_. The structure presented here indicates that inositol polyphosphates can interact specifically with PH domains and therefore lends further support to the idea that at least some inositol phosphates may regulate PH domain phosphoinositide association.

## Results and Discussion

### Addition of IP_6 _effectively competes for binding of PtdIns(3,4)*P*_2 _to C-PH

**Figure 1:**
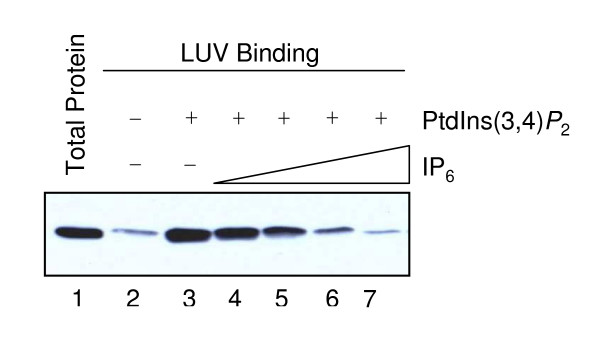
**Effects of IP_6 _on the Binding of C-PH to LUVs containing PtdIns(3,4)*P*_2_**. C-PH binding to LUVs was tested as follows: lane 1, total C-PH added to incubations (0.3 μM); lane 2, C-PH bound to LUVs containing only phosphatidylcholine; lane 3, C-PH bound to LUVs also containing 5 mol% PtdIns(3,4)*P*_2 _; lanes 4–7, as lane 3 with increasing concentrations of IP_6 _(3, 10, 30 and 100 μM).

Using large unilamellar vesicles containing 5 mol% PtdIns(3,4)*P*_2_, we found that simultaneous addition of IP_6 _was able to prevent the binding of C-PH to PtdIns(3,4)*P*_2_. As shown in Figure 1, the binding of C-PH to PtdIns(3,4)*P*_2 _was markedly inhibited by addition of 10 μM IP_6 _and completely abolished by 100 μM IP_6_. Only trace non-specific binding of C-PH to phosphatidylcholine was observed (Figure 1, lane 2). Using a range of IP_6 _concentrations, the apparent IC_50 _of the IP_6 _used was determined to be 7.5 μM.

### Structure of C-PH/ligand complex

**Figure 2:**
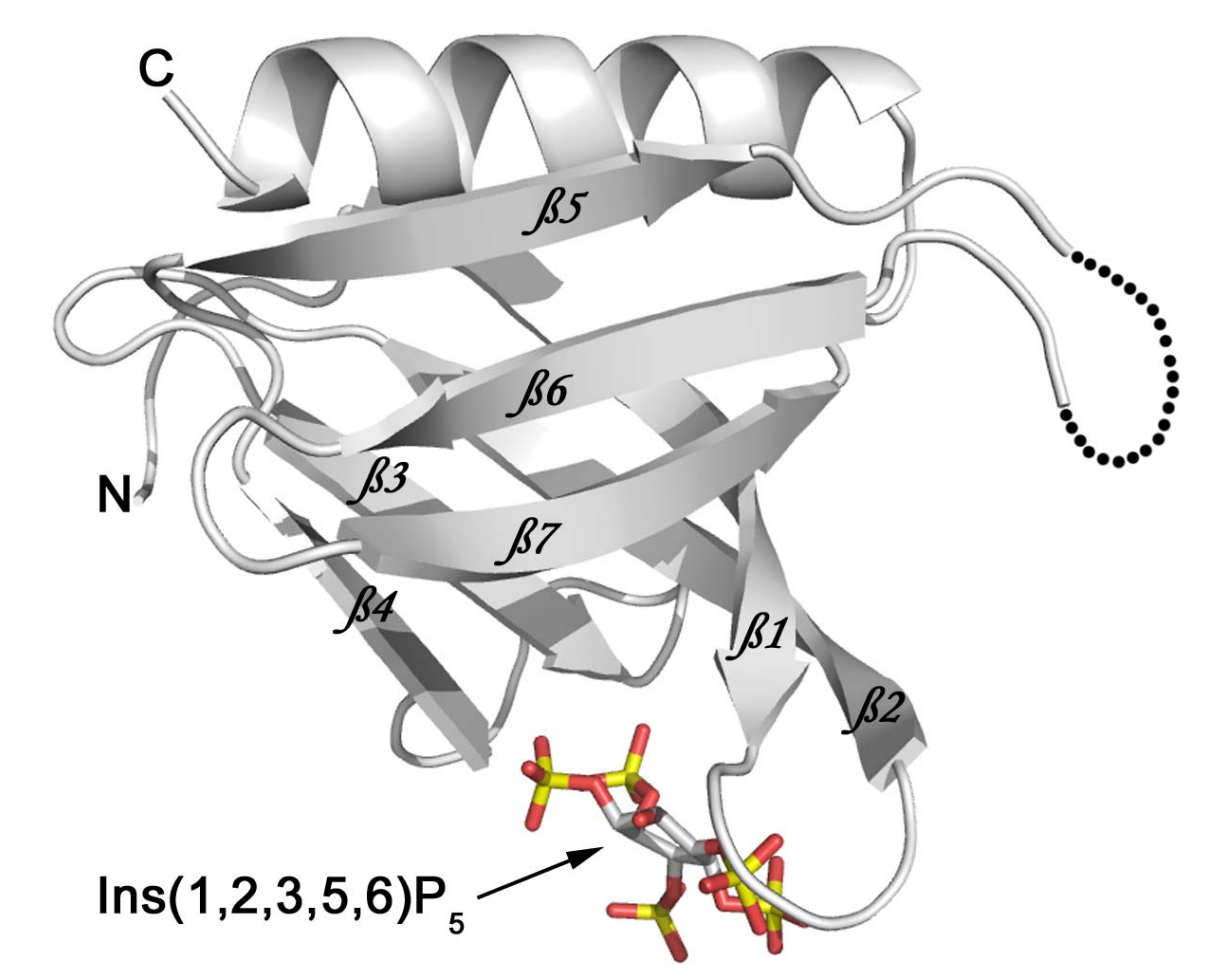
**Overall crystal structure of C-PH bound to Ins(1,2,3,5,6)*P*_5_**. Cartoon representation of C-PH bound to Ins(1,2,3,5,6)*P*_5_. The phosphorous atoms of Ins(1,2,3,5,6)*P*_5 _are colored in yellow while the oxygen atoms are red. The loop region between the 5th and 6th β-strands that could not be built into the model is shown by a broken line.

To investigate further the nature of the *myo*-inositol polyphosphate interaction with C-PH, crystals were prepared using recombinant C-PH and IP_6_, which permitted the determination of a high-resolution crystal structure. Crystals of the complex were grown by the hanging drop vapor diffusion method after incubation of C-PH with 1 mM IP_6 _(see materials and methods). The resulting crystals were of exceptional quality and diffracted to 1.35 Å. Data collection and model refinement statistics are presented in Table 1. The structure was solved by molecular replacement using a search model based on the previously determined C-PH crystal structure solved in the absence of ligand (PDB code: 1ZM0). The final model (Figure 2) was well ordered with the exception of an approximately eight-residue loop region between the fifth and sixth beta strands, corresponding to residues 303–310. Due to the complete disorder of these residues they could not be built into the final model. The overall structure of C-PH containing bound ligand maintained the folding pattern that is characteristic of the PH domain family. Thus, the inositol phosphate ligand was found bound in a loop region of C-PH formed between the first and second beta strands which has been well-characterized in a number of PH domains as the phosphoinositide binding region. Examination of the electron density in this region revealed that the bound D-*myo*-inositol polyphosphate contained only 5 phosphate groups rather than the 6 found in IP_6_. Model building of the different possible D-*myo*-inositol pentakisphosphates into the electron density indicated the identity of the bound ligand to be Ins(1,2,3,5,6)*P*_5_, as no other isomer fits the electron density. The identity of the bound ligand was determined with a high degree of confidence, based on the high-resolution data (1.35 Å), the well-ordered electron density in the binding loop and the fact that only the phosphate at the 2 position on the inositol ring adopts an axial conformation. The presence of Ins(1,2,3,5,6)*P*_5 _was unexpected as a commercial source of IP_6 _was used in the crystallization experiments. However, if the IP_6 _sample contained IP_6 _hydrolysis products this could account for the presence of Ins(1,2,3,5,6)*P*_5 _in the final C-PH structure. This unexpected finding suggests that C-PH has a much higher affinity for Ins(1,2,3,5,6)*P*_5 _than for IP_6_. In support of this view, a separate study [21] showed that the IC_50 _for the inhibition of binding of PtdIns(3,4)*P*_2 _to C-PH by Ins(1,2,3,5,6)*P*_5 _was 2.2 μM. HPLC analysis of the IP_6 _sample used in these studies using a reverse-phase C_18 _column (Vydac 218TP54 RP column) revealed the IP_6 _sample was in fact not pure. Conditions used to generate IP_5_-C-PH co-crystals were mild and therefore not likely to have resulted in conversion of IP_6 _to Ins(1,2,3,5,6)*P*_5_. Regardless of the source, Ins(1,2,3,5,6)*P*_5 _was found tightly bound within the phosphoinositide binding pocket of C-PH and provided important information on the binding of D-*myo*-inositol polyphosphates to PH domains.

**Table 1:** Crystallographic and Data Refinement Statistics

	**C-PH/Ins(1,2,3,5,6)P**_5_
**Date Collection**	
**Space group**	P212121
**Unit-cell parameters (Å)**	a = 32.2, b = 47.7 and c = 64.1 α = β = γ = 90
**No. of molecules in asymmetric unit**	1
**Resolution range (Å)^a^**	32.03 – 1.35 (1.40–1.35)
**Unique reflections**	21 808
**Data Redundancy^a^**	12.21 (7.17)
**Completeness (%)**^a^	97.7 (95.9)
**I/σ(I)^a^**	25.1 (3.9)
**R_merge_(%)^a^**	4.4 (45.4)
**Model and refinement**	
**Resolution range (Å)^a^**	38.26 – 1.35 (1.39–1.35)
**R_work _(%)**	18.0
**R_free _(%)**	20.8
**No. of reflections**	20 722 (19 638 in working set and 1084 in test set)
**Cutoff criterion**	
**No. of amino acid residues/atoms**	99/999
**No. of waters**	125
**r.m.s.d bond lengths (Å)**	0.034
**r.m.s.d bond angles (°)**	3.2
**Average *B *factor (Å^2^)**	23.2
**PDB code**	2I5F

^a^Data for the highest resolution shell are shown in parentheses.

### Analysis of the C-PH/Ins(1,2,3,5,6)*P*_5 _Complex

**Figure 3:**
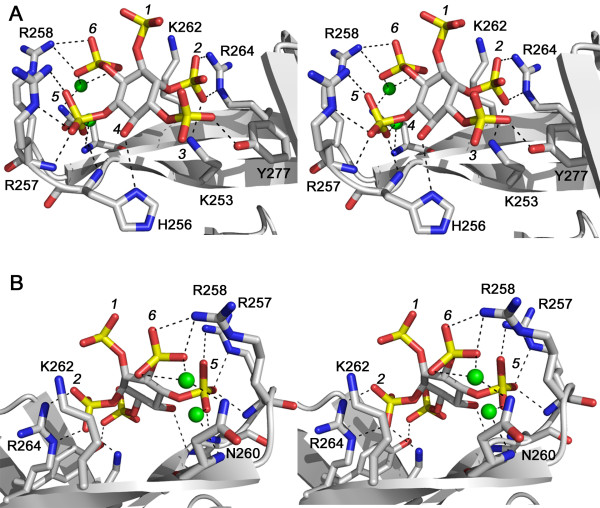
**Interactions between C-PH and Ins(1,2,3,5,6)*P*_5_**. **A. **and **B. **Two stereo views illustrating C-PH binding interactions with Ins(1,2,3,5,6)*P*_5_. Amino acid side chains and backbone atoms from C-PH making specific ligand contacts are indicated. The two water molecules mediating protein-ligand binding are shown as green balls.

The final model of IP_5_-bound C-PH was determined at exceptionally high resolution and consequently the interactions observed between ligand and protein (Figure 3) can be reported with a high degree of certainty. Table 2 lists the specific interactions and distances observed within the crystal structure between Ins(1,2,3,5,6)*P*_5 _and C-PH. Ins(1,2,3,5,6)*P*_5 _was bound to C-PH in the β1–β2 loop region (residues 253–264 and 277), making numerous interactions through all but one of its phosphate groups (Figure 3). The only phosphate group that does not interact with any residues of C-PH is that in the 1-position of *myo*-inositol. Although exposed to solvent, this phosphate remained ordered to the point of generating clear electron density. All amino acids that form stabilizing interactions with Ins(1,2,3,5,6)*P*_5 _are located in the β1–β2 loop region with the exception of Y277 which contributes a hydrogen bond and is situated on the β3 strand of C-PH. The 2-phosphate is the only phosphate that adopts an axial position. This configuration is strongly stabilized through interactions with the side chains of R264 and K253. Unlike the 2-phosphate, the 3-phosphate makes only a single interaction, in this case with Y277. As already mentioned, the 4-phosphate is not present in the structure. However the remaining 4-OH group, which represents the location where the additional phosphate group of IP_6 _would be, does make an interaction with the side chain of H256 at a distance of 3.5 Å. All attempts to model in a phosphate group at this position resulted in steric clashes with H256 and the main chain of residues R257 or G255, depending on the position of the phosphate chosen. In addition, the electron density for H256 is well-ordered indicating that this side chain is not mobile and could not adopt a conformation that would accommodate an additional phosphate group at the 4-position. The 5-phosphate of Ins(1,2,3,5,6)*P*_5 _interacts with main chain atoms of H256, R257 and N260 (water-mediated), as well as the side chain of R257. The final position on the inositol ring is occupied by the 6-phosphate and interacts with the side chain of R258 in addition to the main chain of N260 through a water molecule. C-PH residues shown here to interact with Ins(1,2,3,5,6)*P*_5 _are also known to be involved in binding PtdIns(3,4)*P*_2 _[18]. This demonstrates that Ins(1,2,3,5,6)*P*_5 _competes directly with PtdIns(3,4)*P*_2 _for binding to C-PH. While it is not known exactly how PtdIns(3,4)*P*_2 _interacts with residues in the binding loop, it seems reasonable that two of the phosphates from Ins(1,2,3,5,6)*P*_5 _will directly compete with the phosphates from PtdIns(3,4)*P*_2 _while the remaining phosphates will provide additional stabilizing interactions.

**Table 2:** C-PH/Ins(1,2,3,5,6)*P*_5 _Interactions

**Ins(1,2,3,5,6)*P*_5_**			**C-PH**		
**Position**	**Atom**	Interaction*	**Residue**	**Atom**	**Distance (Å)**

1	O11	-			
	O21	-			
	O31	-			
	O41	-			
					
2	O12	-			
	O22	+	Arg-264	N_η2_	2.7
	O32	-			
	O42	+	Arg-264	N_ε_	2.7
		+	Lys-253	N_ζ_	2.8
					
3	O13	-			
	O23	-			
	O33	-			
	O43	+	Tyr-277	O_η_	2.5
					
4	O14	+	His-256	N_δ1_	3.5
					
5	O15	-			
	O25	+	Arg-257	N_η2_	3.2
	O35	+	His-256	N	3.1
	O45	+	His-256	N	3.2
		+	Arg-257	N	2.7
		+	Arg-257	N_ε_	3.0
					
6	O16	-			
	O26	+	Arg-258	N_η2_	3.1
	O36	-			
	O46	-			

*-, no interaction; +, interaction as shown.

### Comparison of the C-PH/Ins(1,2,3,5,6)*P*_5 _and Grp1/Ins(1,3,4,5,6)*P*_5 _structures

**Figure 4:**
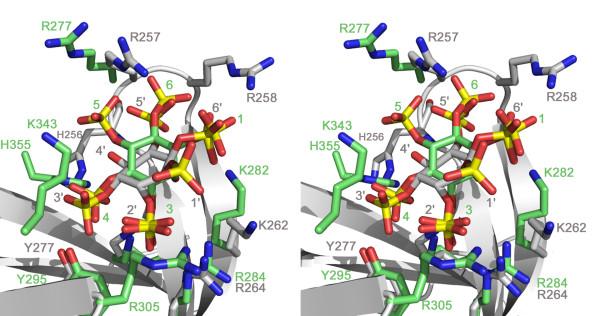
**Structural comparison of the Grp1/Ins(1,3,4,5,6)*P*_5 _and C-PH/Ins(1,2,3,5,6)*P*_5 _structures**. Stereo view of structurally superimposed Grp1/Ins(1,3,4,5,6)*P*_5 _and C-PH/Ins(1,2,3,5,6)*P*_5_. Grp1 and ligand, green; C-PH and ligand, grey. For simplicity a ribbon diagram for C-PH is shown.

Although the structure presented here represents the first high resolution structure of Ins(1,2,3,5,6)*P*_5 _bound to a PH domain, the structure of another PH domain complexed with Ins(1,3,4,5,6)*P*_5 _has been reported [22]. The PH domain of Grp1 binds 3-phosphoinositides allowing the protein to participate in regulating the actin cytoskeleton in response to phosphoinositide-3-kinase signaling pathways. As such, the structure of the PH domain of Grp1 complexed with Ins(1,3,4,5,6)*P*_5 _represents another instance in which specific interactions of an inositol phosphate and the phosphoinositide binding cleft of a PH domain might be expected to regulate the association of the PH domain with a specific phosphoinositide. Comparison of the Grp1 PH domain complexed with Ins(1,3,4,5,6)*P*_5 _(PDB code: 1FHW) with that of C-PH bound to Ins(1,2,3,5,6)*P*_5_, revealed some intriguing similarities and differences (Figure 4). Although both D-*myo*-inositol pentakisphosphates are bound in the β1–β2 loop region, they adopt slightly different orientations with respect to their inositol rings (Figure 4). Remarkably, despite this difference in the overall orientation of the inositol ring, four of the five phosphate groups from each ligand occupy overlapping positions in the β1–β2 binding cleft. The 2-phosphate of Ins(1,2,3,5,6)*P*_5 _occupies the same position as the 3-phosphate from Ins(1,3,4,5,6)*P*_5_. In both structures, the phosphate group at this position interacts with a highly conserved arginine residue (R264 in C-PH and R284 in Grp1). The arginine at this position is conserved amongst a number of PH domains including that of Bruton's tyrosine kinase (Btk), in which its mutation to either histidine or proline causes the hereditary immune disease X-linked agammaglobulinaemia [23]. In the crystal structure of the Btk PH domain bound to Ins(1,3,4,5)*P*_4_, it was shown that the conserved arginine (R28) interacts with the 3-phosphate, suggesting that mutation of this residue decreases the affinity of the PH domain for its natural ligand, PtdIns(3,4,5)*P*_3 _[24].

The second overlapping phosphate position is occupied by the 6 and 1-phosphates of Ins(1,2,3,5,6)*P*_5 _and Ins(1,3,4,5,6)*P*_5_, respectively. In the C-PH structure, the 6-phosphate interacts with R258, a residue that is not conserved in the Grp1 PH domain. The corresponding 1-phosphate of Ins(1,3,4,5,6)*P*_5 _interacts with K282 in the Grp1 PH domain which is equivalent to K262 in C-PH, although this residue does not interact with the 6-phosphate of Ins(1,2,3,5,6)*P*_5 _due to the location of its side chain. The 5-phosphate of Ins(1,2,3,5,6)*P*_5 _and the 6-phosphate of Ins(1,3,4,5,6)*P*_5 _occupy the third overlapping position within the phosphoinositide-binding pocket. The 5-phosphate is in position to interact with the main chains of H256, R257 and N260 as well as the side chain of R257 in C-PH. Although this arginine is conserved in the Grp1 PH domain (R277), it does not interact with the 6-phosphate, in fact the 6-phosphate makes less optimal interactions with main chain residues compared to those observed in the C-PH structure. In the fourth and final overlapping position, the 3-phosphate of Ins(1,2,3,5,6)*P*_5 _and the 4-phosphate of Ins(1,3,4,5,6)*P*_5 _both interact with a conserved tyrosine at position 277 in C-PH and 295 in the Grp1 PH domain. In addition to this, the 4-phosphate makes further stabilizing contacts with the side chains of H355 and K273, interactions that are not observed in the C-PH/Ins(1,2,3,5,6)*P*_5 _structure.

**Figure 5:**
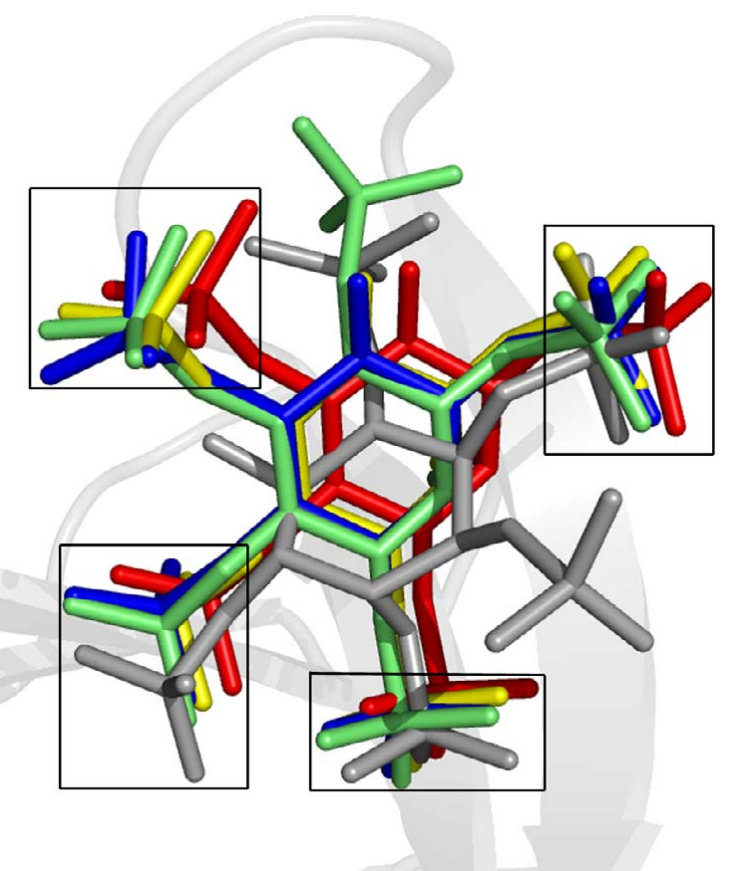
**Conserved binding positions of inositol phosphates to PH domains**. Superimposed inositol phosphates in complex with PH domains from five different proteins: DAPP1/PHISH-Ins(1,3,4,5)*P*_4_, red, 1FAO; Grp1-Ins(1,3,4,5)*P*_4_, yellow, 1FGY; ARNO-Ins(1,3,4,5)*P*_4_, blue, 1U27; Grp1-Ins(1,3,4,5,6)*P*_5_, green, 1FHW; C-PH-Ins(1,2,3,5,6)*P*_5_, gray, 2I5F. The four conserved phosphate binding positions are boxed.

From this structural comparison it can be seen that in both cases the D-*myo*-inositol pentakisphosphate ligands interact with conserved side chains that would be involved in binding phosphoinositides. This suggests that the D-*myo*-inositol pentakisphosphates would directly compete with phosphoinositides for binding to the PH domains. In addition to this, it was found that four out of the five phosphates occupy overlapping positions within the binding cleft despite the differences in orientation and even stereochemistry. This overlap in phosphate positions is not simply coincidence, since a structural alignment of various PH domains solved in complex with either Ins(1,3,4,5)*P*_4 _or the two D-*myo*-inositol pentakisphosphates used in the comparison above reveals that the overlapping positions are conserved in these structures as well (Figure 5). The mode of interaction observed in our structure is therefore consistent with the possibility that *myo-*inositol polyphosphates could act to regulate PH domain-phosphoinositide associations by directly competing with phosphoinositides for binding to PH domains.

### D-*myo*-inositol pentakisphosphates as signaling molecules

The physiological significance of the interaction reported here with respect to platelet signaling pathways is currently unknown and calls for further careful examination. With the exception of IP_3_, inositol phosphates have only been thought to function with minor roles as second messengers [25]. However, as more information becomes available it appears as though these molecules have a much broader role in signaling then was originally appreciated [26,27]. The most well-characterized inositol phosphate second messenger is IP_3_, produced by phospholipase C-mediated hydrolysis of PtdIns(4,5)*P*_2_. In addition to IP_3_, it has also been proposed that Ins(1,3,4,5)*P*_4 _plays a role in the regulation of cellular Ca^+2 ^fluxes [27]. The IP_5 _family of D-*myo*-inositol pentakisphosphates represents one of the most abundant forms of inositol phosphates present in mammalian cells. Although six possible isomers exist, Ins(1,3,4,5,6)*P*_5 _represents the predominant form observed in mammalian cells, including platelets [27-30]. Other IP_5 _isomers including Ins(1,2,4,5,6)*P*_5 _and Ins(2,3,4,5,6)*P*_5 _have been detected in a number of cell lines [29,31], demonstrating that these molecules are indeed present in mammalian cells and as such could play roles in regulating biological functions. In agreement with this, several recent studies have provided evidence suggesting specific roles for IP_5 _isomers ranging from the regulation of chromatin remodeling [32,33] to *Salmonella *pathogenesis [34,35]. In further support of this idea, Ins(1,3,4,5,6)*P*_5 _was recently shown to be capable of promoting apoptosis and to possess antiangiogenic and antitumor effects as a result of its ability to inhibit the phosphoinositide 3-kinase/Akt signaling pathway [36,37]. This inhibition was shown to be a consequence of Ins(1,3,4,5,6)*P*_5 _competing with PtdIns(3,4,5)*P*_3 _for binding to the protein kinase B (Akt) PH domain, ultimately preventing the phosphorylation and activation of Akt. Based on these intriguing results the authors have proposed that Ins(1,3,4,5,6)*P*_5 _and potentially other inositol phosphates can be used as experimental or possibly therapeutic tools that interfere with the binding of PH domains involved in signaling pathways. Inositol pyrophosphates, specifically IP_7 _and IP_8_, have also been shown to act as signaling molecules that regulate PH domain interactions with phosphoinositides [38]. In this study IP_7 _and IP_8 _were shown to compete with PtdIns(3,4,5)*P*_3 _for binding to several PH domain-containing proteins in Dictyostelium, resulting in an effect on chemotaxis. As the body of evidence continues to grow, it appears that PH domains will emerge as versatile domains capable of mediating interactions with a range of different ligands.

It therefore seems likely that as more studies are aimed at examining the roles of IP_5 _isomers, it will become apparent that this family of inositol phosphates is involved in signaling pathways that affect a range of physiological processes. The identification of PH domains capable of interacting specifically with the various IP_5 _isomers will aid in characterizing the roles of the latter. However, we are not aware of any report of the presence of Ins(1,2,3,5,6)*P*_5 _in platelets or other cells at the present time. It will therefore be important to investigate the effects of other IP_5 _isomers on the binding of pleckstrin C-PH domain to PtdIns(3,4)*P*_2_. It has not escaped our attention that in addition to acting as antagonists of pleckstrin binding by specific phosphoinositides, *myo-*inositol polyphosphates with high affinities for C-PH could act as regulators of the interaction of pleckstrin with other proteins.

## Conclusion

In the work presented here, the addition of commercial IP_6 _was shown to compete with PtdIns(3,4)*P*_2 _for binding to C-PH. Since Ins(1,2,3,5,6)*P*_5 _was found bound to C-PH in the crystal structure it would appear that the inhibitory effects observed in the binding studies were due to contamination of IP_6 _with Ins(1,2,3,5,6)*P*_5_, which binds to C-PH with a higher affinity. Regardless of its source, Ins(1,2,3,5,6)*P*_5 _binds specifically in the phosphoinositide binding cleft of C-PH making numerous interactions with residues known to be involved in binding PtdIns(3,4)*P*_2_[18]. In a structural comparison with the Grp1 PH domain bound to Ins(1,3,4,5,6)*P*_5 _it was observed that despite differences in their arrangement about the inositol ring, four out of the five phosphate groups from these two ligands occupy conserved positions. This structural analysis, in combination with other recently published data discussed above, suggests that *myo*-inositol pentakisphosphates could act to regulate PH domain-phosphoinositide interactions by directly competing for binding to these domains. It is also possible that *myo*-inositol pentakisphosphates could play roles in signaling pathways by acting as inducers of protein-protein interactions.

## Methods

### C-PH binding assays

The binding assays were performed as described in [19]. Briefly, 0.3 μM recombinant C-PH was incubated with large unilamellar vesicles (LUVs) consisting of phosphatidylcholine supplemented with 5 mol% PtdIns(3,4)*P*_2_. Simultaneous additions of IP_6 _(Aldrich) (0–100 μM) were used to inhibit the binding of C-PH to the PtdIns(3,4)*P*_2_. LUVs with bound protein were isolated by ultracentrifugation, analyzed by SDS-PAGE using 10% acrylamide and immunoblotted with antibody to the C-terminal 13 residues of pleckstrin. A logit plot was used to calculate the apparent IC_50 _of IP_6 _(y = 100[1 + (x/IC_50_)^s^]^-1^, where y is the % of added C-PH bound at the inhibitor concentration x and s is a slope factor).

### Protein expression and purification

C-PH (pleckstrin amino acids 240–350) used for LUV binding studies was expressed and purified as described previously [19,20]. For crystallography, C- and N-terminal residues previously found to lack structure [20] were omitted. Thus, the carboxy terminal PH domain (amino acids 243–347) of human pleckstrin was expressed and purified as a hexahistidine fusion protein in *E. coli *BL21(DE3) cells using the pDEST17 expression vector (Invitrogen). Cells were grown in standard LB medium supplemented with 10 mg ml^-1 ^ampicillin at 310 K with shaking (225 rev min^-1^) until the light absorbance at 600 nm reached 0.5. Protein expression was then induced using 0.1 mM IPTG and the incubation temperature was lowered to 300 K. After a 5-hour induction period cells were harvested by centrifugation at 4 000 rpm for 10 minutes at a temperature of 277 K. Each 1L cell pellet was then resuspended in 8 ml of 1× phosphate buffered saline (PBS) and centrifuged at 3 500 rpm for 10 minutes at 277 K. The resulting cell pellets were then frozen in liquid nitrogen and stored at 193 K. Prior to cell lyses using high pressure, cell pellets (2 L) were resuspended to a final volume of 35 ml using nickel A buffer (20 mM Hepes pH 7.5, 0.5 M NaCl, 0.06% LDAO, 2 mM β-mercaptoethanol and 10 mM imidazole). Following cell lyses samples were centrifuged at 20 000 rpm for 40 minutes at 277 K and the resulting supernatant was applied to a HiTrap Nickel affinity column (Amersham Biosciences). The C-PH was eluted from the column with 210 mM imidazole following sequential washes with 15 and 30 mM imidazole which was accomplished by mixing appropriate volumes of nickel A buffer and nickel B buffer (20 mM Hepes pH 7.5, 0.5 M NaCl, 0.06% LDAO, 2 mM β-mercaptoethanol and 300 mM imidazole). The protein sample was then diluted in S-A buffer (10 mM Hepes pH 7.5, 1 mM dithiothreitol and 1 mM EDTA) to lower the salt concentration to approximately 75 mM. This sample was then applied to a HiTrap SP Sepharose HP ion exchange column (Amersham Biosciences) to further purify the protein from any remaining contaminants. The C-PH was subsequently eluted using a gradient of increasing salt concentration generated by the application of S-B buffer (10 mM Hepes pH 7.5, 1 mM dithiothreitol, 1 mM EDTA and 0.5 M NaCl). The hexahistidine tag was removed from the C-PH by cleavage with TEV protease. This results in four residues (Gly, Ser, Phe and Thr) being left N-terminal to the first residue in C-PH. Of these four amino acids only the phenylalanine and threonine residues could be reliably built into the available electron density. In order to purify C-PH from the cleaved hexahistidine tag and the TEV protease the digested sample was re-applied to the HiTrap SP Sepharose column and purified as described above. The resulting C-PH sample was buffer exchanged into the final crystallization buffer (5 mM pottasium glutamate, 120 mM sodium glutamate, 20 mM Hepes pH 7.5, 2.5 mM EDTA, 2.5 mM EGTA and 3.15 mM MgCl_2_) using a HiPrep 26/10 Desalting column (Amersham Biosciences). The resulting protein sample was concentrated to 4.0 mg ml^-1 ^as determined by the Bradford assay, using a centrifugal filter. SDS-PAGE analysis indicates that C-PH prepared by this method is greater then 95% pure.

### Crystallization and data collection of the C-PH/Ins(1,2,3,5,6)*P*_5 _complex

C-PH (2.5 mg ml^-1^) was crystallized in the presence of 1 mM IP_6 _(Aldrich) using the hanging drop vapour diffusion method under the following conditions. A 2 μl drop containing 1 μl of C-PH (2.5 mg ml^-1^) and 1 mM IP_6 _in 5 mM potassium glutamate, 120 mM sodium glutamate, 20 mM Hepes pH 7.5, 2.5 mM EDTA, 2.5 mM EGTA and 3.15 mM MgCl_2 _and 1 μl of 0.1 M Bis-Tris pH 6.5 and 28% polyethylene glycol 2 000 monomethyl ether was suspended over 500 μl of 0.75 M ammonium sulfate and incubated at 293 K. Rectangular rod-shaped crystals grown in these conditions were harvested and crushed to generate micronuclei that were subsequently used to streak seed into the same crystallization condition as well as slight variations of this condition. This resulted in large rectangular rod-shaped crystals with dimensions of approximately 400 × 100 × 100 μm. The crystal used to collect data was grown in 0.1 M Bis-Tris pH 6.5 and 28% polyethylene glycol 2 000 monomethyl ether. Prior to cryocooling in a nitrogen cold stream, the crystal was soaked for approximately 10 seconds in a cryoprotectant containing 0.1 M Bis-Tris pH 6.5, 28% polyethylene glycol 2 000 monomethyl ether and 17% glycerol. Both high (1.35 Å) and low (1.5 Å) resolution data sets were collected independently for this crystal and then merged to yield a single data set. The data was collected at a wavelength of 1.0 Å at beamline X25 of the Brookhaven National Laboratory using a ADSC Q315 CCD x-ray detector and processed using the HKL2000 program suite [39].

### Structure determination and model refinement

The crystal structure of the C-PH in complex with Ins(1,2,3,5,6)*P*_5 _was solved by molecular replacement using the program *MOLREP *[40]. The search model used in molecular replacement was the crystal structure of the C-PH (PDB code 1ZM0), which was solved in the absence of any ligand. Iterative cycles of model building and refinement were carried out using the programs *WinCoot *[41] and *Refmac5 *[42,43] respectively. All figures describing protein structures presented were generated using the molecular graphics program *PyMol *[44].

## Authors' contributions

SGJ purified C-PH, crystallized the complex, collected and processed crystallographic data, refined the structure and prepared the manuscript. YZ purified C-PH and performed the binding studies. RJH participated in designing the study and preparing the manuscript. MSJ was involved in designing and overseeing the study as well as preparing the manuscript. All authors have read and approved the final manuscript.
